# Impact of sugarcane irrigation on malaria vector *Anopheles* mosquito fauna, abundance and seasonality in Arjo-Didessa, Ethiopia

**DOI:** 10.1186/s12936-020-03416-0

**Published:** 2020-09-22

**Authors:** Assalif Demissew, Dawit Hawaria, Solomon Kibret, Abebe Animut, Arega Tsegaye, Ming-Cheih Lee, Guiyun Yan, Delenasaw Yewhalaw

**Affiliations:** 1 Department of Medical Laboratory Sciences, College of Medicine and Health Sciences, Ambo University, Ambo, Ethiopia; 2 Aklilu Lemma Institute of Pathobiology, Addis Ababa University, Addis Ababa, Ethiopia; 3 Yirgalem Hospital Medical College, Yirgalem, Ethiopia; 4 School of Medical Laboratory Sciences, Faculty of Health Sciences, Jimma University, Jimma, Ethiopia; 5 College of Natural Science, Department of Biology, Jimma University, Jimma, Ethiopia; 6 Program in Public Health, University of California at Irvine, Irvine, CA 92697 USA; 7 Tropical and Infectious Diseases Research Center (TIDRC), Jimma University, Jimma, Ethiopia

**Keywords:** Malaria, Irrigation, *Anopheles* mosquitoes, Vector density, *Anopheles amharicus*, Ethiopia

## Abstract

**Background:**

Despite extensive irrigation development in Ethiopia, limited studies assessed the impact of irrigation on malaria vector mosquito composition, abundance and seasonality. This study aimed to evaluate the impact of sugarcane irrigation on species composition, abundance and seasonality of malaria vectors.

**Methods:**

Adult *Anopheles* mosquitoes were collected using CDC light traps from three irrigated and three non-irrigated clusters in and around Arjo-Didessa sugarcane irrigation scheme in southwestern Ethiopia. Mosquitoes were surveyed in four seasons: two wet and two dry, in 2018 and 2019. Mosquito species composition, abundance and seasonality were compared between irrigated and non-irrigated clusters. *Anopheles* mosquitoes were sorted out to species using morphological keys and molecular techniques. Chi square was used to test the relationships between *Anopheles* species occurrence, and environmental and seasonal parameters.

**Results:**

Overall, 2108 female *Anopheles* mosquitoes comprising of six species were collected. Of these, 92.7% (n = 1954) were from irrigated clusters and 7.3% (n = 154) from the non-irrigated. The *Anopheles gambiae complex* was the most abundant (67.3%) followed by *Anopheles coustani* complex (25.3%) and *Anopheles pharoensis* (5.7%). PCR-based identification revealed that 74.7% (n = 168) of the *An. gambiae* complex were *Anopheles arabiensis* and 22.7% (n = 51) *Anopheles amharicus*. The density of *An. gambiae* complex (both indoor and outdoor) was higher in irrigated than non-irrigated clusters. The overall anopheline mosquito abundance during the wet seasons (87.2%; n = 1837) was higher than the dry seasons (12.8%; n = 271).

**Conclusion:**

The ongoing sugarcane irrigation activities in Arjo-Didessa created conditions suitable for malaria transmitting *Anopheles* species diversity and abundance. This could drive malaria transmission in Arjo-Didessa and its environs in both dry and wet seasons. Currently practiced malaria vector interventions need to be strengthened by including larval source management to reduce vector abundance in the irrigated areas.

## Background

Irrigation-based agriculture has been largely promoted to alleviate poverty and improve economic growth in Africa [1]. However, existing evidence shows that irrigation might increase the risk of vector-borne diseases such as malaria [2–5]. Man-made environmental modifications and expansion of unplanned water development schemes could enhance mosquito breeding and sustain malaria transmission [2–5]. In Ethiopia, where malaria is a major cause of morbidity and hospital admissions [6], irrigation activities may contribute to increased risk of the disease. Irrigations can enhance malaria transmission by increasing the number and diversity of mosquito-breeding habitats (e.g., poorly managed irrigation canals and canal seepages) that can increase vector composition, density and longevity. This can ultimately increase risk of malaria and extend the duration of malaria transmission in irrigation areas in Ethiopia where the disease is seasonal and unstable [7–9].

Previous studies indicate higher malaria risk close to dam and irrigation schemes compared to communities living further away [9–11]. In northern Tanzania, a 4-fold increase in the density of *Anopheles arabiensis* and risk of malaria was documented in rice irrigation fields than in non-irrigated savannah villages [11]. In Ghana, higher larval and adult anopheline densities were observed in irrigated areas compared to non-irrigated areas in the rainy and dry seasons [12]. Similarly, in Ethiopia, villages practicing irrigated agriculture were shown to have increased malaria vector abundance [13], risk of malaria infection [14] and mosquito density [9, 10] compared to non-irrigated villages. However, some studies indicate that irrigated sugarcane cultivation resulted in water pooling but did not produce more vectors [11]. In general, unlike areas where malaria is stable, irrigation practices in areas of unstable malaria could affect vector abundance and lead to increased transmission [2].

Although several studies reported an increase in mosquito density and malaria transmission associated with rice irrigation [2], little is known about the impact of sugarcane irrigation on malaria transmission in Africa [11]. Available data from cross-sectional studies failed to depict trends of temporal malaria vector dynamics at least in the two major seasons: dry and wet. Studies that evaluate the impact of sugarcane irrigation on malaria mosquito dynamics are scarce in Ethiopia despite the country’s unprecedented expansion in irrigation practices.

Thus, it was deemed necessary to evaluate the current impact of sugarcane irrigation on vector distribution, abundance and seasonality pattern in a way to suggest vector control interventions and inform public health professionals [15]. Furthermore, knowledge of the dynamics and behaviour of local *Anopheles* mosquitoes may help devise control tools to achieve malaria elimination goal [16]. This study aimed to assess the impact of Arjo-Didessa sugarcane irrigation on species composition, seasonality and abundance of *Anopheles* mosquitoes. The study tests the hypothesis that irrigation increases mosquito abundance both during the dry and wet seasons of the year.

## Methods

### Study setting

**Fig. 1:**
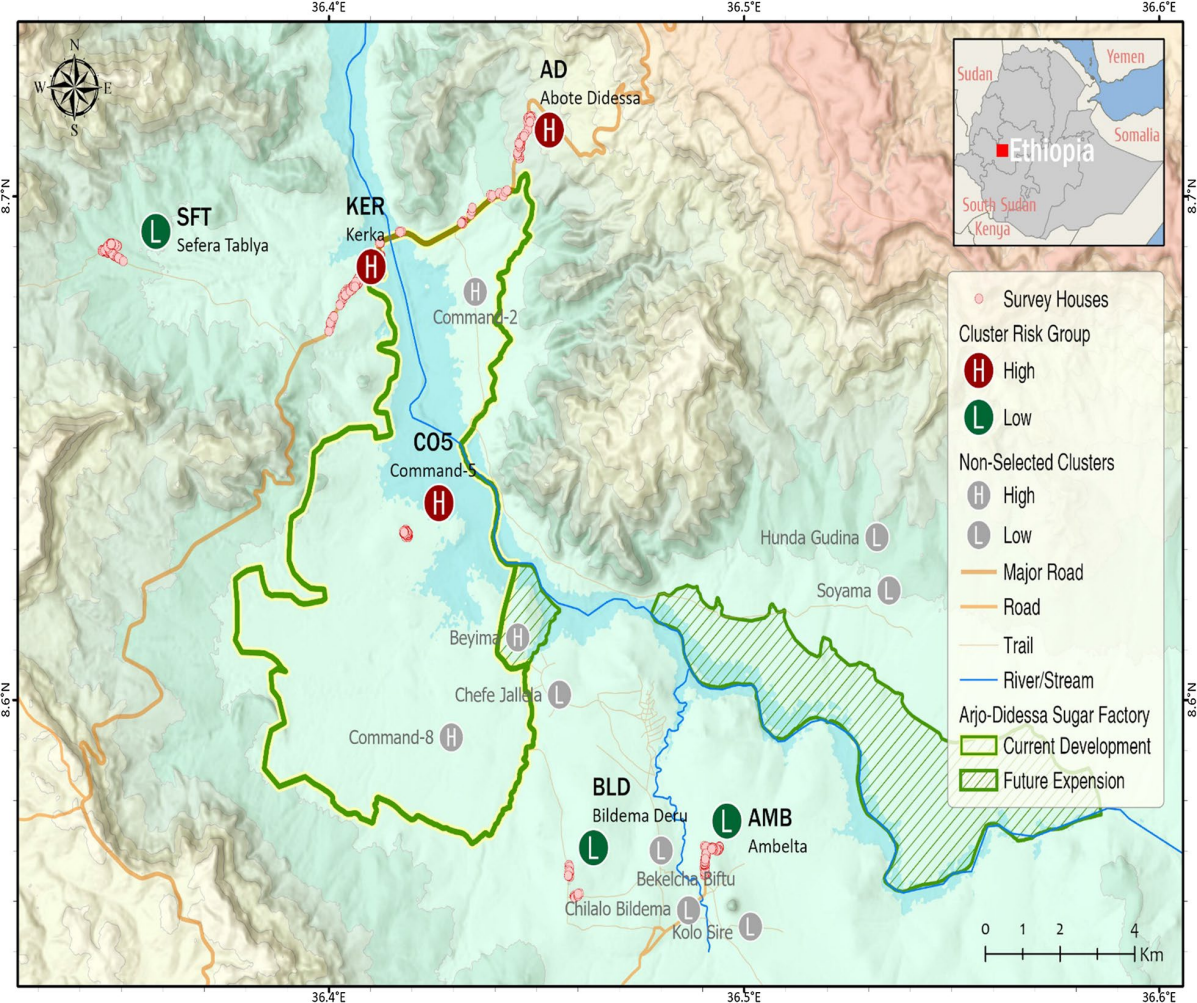
Map of Arjo-Didessa sugar cane irrigated clusters (Command-5, Kerka and Abote-Didessa) and non-irrigated clusters (Ambelta, Bildema Deru and Sefera Tabiya), Southwest Ethiopia. NB: H, Irrigated Clusters; L, Non-irrigated Clusters

The study was conducted at Arjo-Didessa sugarcane irrigation scheme and its surroundings located at 395 km southwest of the capital, Addis Ababa, Ethiopia (Fig. 1). Six study clusters were randomly selected out of 15 clusters from three districts: Jimma Arjo district (Abote Didessa), Bedele District (Command 5, Bildema Deru and Ambelta) and Dabo Hana district (Kerka and Sefera Tabiya). They were selected on the basis of their proximity to the irrigation activities. The irrigation clusters (within and about 3 km from the irrigation area) were Command-5, Kerka and Abote-Didessa while the non-irrigated (4–10 km from the irrigation area) were Ambelta, Bildema Deru and Sefera Tabiya. The shortest distance between the irrigated and the non-irrigated clusters was about 4–5 km and selection of the study clusters was by assuming an average *Anopheles* mosquito flight range of 3 km to control overlap/contamination of mosquitoes flying from the irrigated to the non-irrigated and vice versa. A cluster is defined as an area that has 150–200 households. Both clusters had similar eco-topography. Entomological surveys were conducted from January 2018 to August 2019 in four seasons: two wet seasons and two dry seasons in the six clusters.

The districts have a total population of 215,288 and the study clusters population was 50,000. The great majority of the population depends on subsistence farming. People in the non-irrigated clusters (Ambelta, Bildema Deru, Sefera Tabiya) commonly raise cattle and cultivate mixed crops and cereals, including sorghum, rice, corn/maize, peanut and vegetables during the rainy season. Among the irrigated clusters, Command-5 is at the centre of sugarcane irrigation where farm employees live, while residents of Kerka and Abote-Didessa border the sugarcane irrigation area (Fig. 1), and use mixed farming, often planting sugarcane in their backyards. The altitude of the area ranges from 1300 to 2280 m above sea level with mean annual rainfall of 1477 mm. The irrigation area and its surroundings are known to be malarious [17]. It was formerly a wildlife sanctuary (Didessa wildlife sanctuary), but since 2006 changed to a state-owned sugarcane plantation development to supply the sugar factory. It is one of the biggest sugar development projects in Ethiopia, covering about 5000 ha of land with future expansion plan of 80,000 ha.

### Mosquito sampling and processing

Adult *Anopheles* mosquitoes were collected using standard Centers for Disease Prevention and Control (CDC) light traps (Model: John W. Hock CDC Light trap 512, USA) from eight randomly selected houses in each of the six clusters. At each sampling night, 16 CDC light traps were installed in each cluster. Eight light traps were placed indoors inside bedrooms at about 1.5 m above the floor near the foot end of a person sleeping under long-lasting insecticide-treated net and another eight installed outdoors at about 5 m from the same house used for indoor collection. The traps were kept running from 18:00 to 06:00 h. A total of 192 trapping nights were spent indoors and outdoors in each cluster during the study period.

After 06:00, the CDC light traps were labelled with identifier, collected and transported to the field laboratory for processing. Live and dead mosquitoes were retrieved by mechanical aspirator from collection bags and live mosquitoes were killed using chloroform (99.8% Trichloromethane). Female *Anopheles* mosquitoes were sorted and identified morphologically under dissecting microscope to species using standard key [18]. Abdominal status of the mosquitoes was determined under dissecting microscope as unfed, freshly fed, half-gravid, or gravid. Culicine and male anopheline mosquitoes were also retrieved by aspirator from the bags, counted and recorded. Each female *Anopheles* mosquito was preserved individually in labelled Eppendorf tube over silica gel and stored for further processing. Sample processing was done at Arjo-Didessa International Centre of Excellence for Malaria Research (ICEMR) Laboratory, Ethiopia.

### Identification of *Anopheles gambiae* complex species

Among the total 1418 *An. gambiae* sensu lato (*s.l*.) collected during the survey, some 225 (~ 16%) were randomly selected and identified to species by using species-specific polymerase chain reaction (PCR) assay at the Molecular Biology Laboratory of Tropical and Infectious Diseases Research Centre (TIDRC), Jimma University, Ethiopia. Briefly, genomic DNA was extracted using DNA extraction kit (Qiagen, Sigma Aldrich, USA) from legs and wings of each mosquito. PCR assay was carried out according to the methods of Scott et al. [19] using species specific primers. After PCR amplification was complete, the amplicon was loaded on 1.5% agarose gel stained with ethidium bromide and run for gel electrophoresis. *Anopheles arabiensis* from Sekoru insectary colony of Jimma University was used as a positive control.

### Data analysis

Data entry and analysis was made using Microsoft Excel (Version 2016, Microsoft Corp, USA) and IBM SPSS version 20.0 (SPSS Inc., Chicago, IL, USA) statistical software packages and had been summarized with frequencies (n) and percentages (%) by species, season and irrigation levels. Chi square (χ2) test was used to compare mosquito variation by irrigation level and season and the test was assumed significant at a *p* value of less than 0.05. Indoor and outdoor mosquito density for each species per household was calculated as:

$${\text{``D }} = {\text{ n}}/{\text{trap}} - {\text{night''}}$$

where ‘D’ is density for individual mosquito species and ‘n’ is the number of mosquitoes for every species, ‘trap-night’ represents the trapping night spent in each house of all clusters. Note that the frequency of collection, the number of traps used and the number of nights spent in each season and in each cluster was similar.

Shannon diversity index was calculated to compare species richness and diversity in the irrigated and non-irrigated clusters. Shannon’s diversity index (H) was determined as follows:

$${\text{H}} = \sum \left[ {\left( {\text{pi}} \right) \times { \ln }\left( {\text{pi}} \right)} \right],$$

where pi is proportion of total number of samples represented by species i out of the total number of samples.

### Ethical considerations

Ethical clearance was obtained from the Institutional Review Board (IRB) of Aklilu Lemma Institute of Pathobiology, Addis Ababa University, Ethiopia (Ref. *No. ALIPB/IRB/012/2017/18*) and National Ethics Review Committee (NERC), Ethiopia. Permission was also obtained from East Wollega and Buno Bedele Zonal Health Offices, Oromia Regional State, Ethiopia. Verbal consent was obtained from household owners to set CDC light traps.

## Results

### Species composition of *Anopheles gambiae* complex

Among the 225 *An. gambiae s.l.* tested for species identification using PCR, 74.7% (n = 168) were found to be *An. arabiensis*, 22.7% (n = 51) *Anopheles amharicus* (formerly known as *Anopheles quadriannulatus* B) and the remaining 2.6% (n = 6) samples were not amplified.

### *Anopheles* species composition and abundance

Overall, 2108 (38.8%) anopheline and 3326 (61.2%) culicine mosquitoes were collected from the six clusters during the study period. Among the 2108 anopheline mosquitoes, 92.7% (n = 1954) were from the irrigated clusters and 7.3% (n = 154) from the non-irrigated (control) clusters (Table 1). Six *Anopheles* species were identified in the irrigated clusters while only four species recorded in the non-irrigated cluster. PCR based analyses of sub-samples of *An. gambiae s.l.* from non-irrigated clusters revealed only *An. arabiensis*.

**Table 1:** Composition and abundance of *Anopheles* species in sugarcane irrigated and non-irrigated areas of Arjo-Didessa irrigation scheme, Southwest Ethiopia, 2018–2019

Cluster	*An. coustani* n (%)	*An. funestus* n (%)	*An. gambiae s.l* n (%)	*An. pharoensis* n (%)	*An. squamosus* n (%)	Total n (%)
Irrigated clusters						
Command 5	267 (22.2)	4 (0.3)	849 (70.6)	82 (6.8)	0 (0.0)	1202 (100)
Abote Didessa	6 (15.0)	3 (7.5)	31 (77.5)	0 (0.0)	0 (0.0)	40 (100)
Kerka	208 (29.2)	4 (0.6)	467 (65.6)	16 (2.2)	17 (2.4)	712 (100)
Non-irrigated clusters						
Ambelta	28 (28.0)	0 (0.0)	49 (49.0)	22 (22)	1 (1.0)	100 (100)
Bildema Deru	24 (64.9)	0 (0.0)	6 (16.2)	0 (0.0)	7 (18.9)	37 (100)
Sefera Tabiya	1 (5.9)	0 (0.0)	16 (94.1)	0 (0.0)	0 (0.0)	17 (100)
Total, n (%)	534 (25.33)	11 (0.52)	1418 (67.27)	120 (5.69)	25 (1.19)	2108 (100)

*Anopheles gambiae s.l.* (n = 1418; 67.27%) was the most abundant species followed by *Anopheles coustani* complex (n = 534; 25.33%), *Anopheles pharoensis* (n = 120; 5.69%), *Anopheles squamosus* (n = 25; 1.19%) and *Anopheles funestus* group (n = 11; 0.52%). The *Anopheles* mosquito abundance in the irrigated clusters was significantly greater than in the non-irrigated clusters (χ2 = 61.404, df = 4, *P *< 0.001). *Anopheles gambiae s.l,* was more abundant in the irrigated clusters (n = 1347; 95%) than in the non-irrigated (n = 71; 5%). Similarly, *An. coustani* complex, *An. pharoensis* and *An. squamosus* showed higher abundance in the irrigated clusters relative to the non-irrigated clusters.

### Mosquito density and seasonality

Majority of mosquitoes were collected from outdoor (n = 1247, 59.2%) than indoor (n = 861, 40.8%) and the difference was statistically significant (χ2 = 188.07, df = 4, *P *< 0.001) (Table 2). Density of *An. gambiae s.l.* was higher in the irrigated than in the non-irrigated clusters. In the irrigated clusters, *An. gambiae s.l.* showed a slightly higher density outdoors (3.55 mosquitoes per trap per night) than indoors (3.46 mosquitoes per trap per night). In contrast, the density of *An. gambiae s.l.* in the non-irrigated clusters was slightly higher indoors (0.21 mosquito per trap per night) than outdoors (0.16 mosquito per trap per night). Other *Anopheles* species generally showed a higher density outdoors than indoors in both irrigated and non-irrigated clusters.

**Table 2:** Indoor and outdoor anopheline mosquito density in irrigated and non-irrigated clusters of Arjo-Didessa irrigation scheme, Southwestern Ethiopia, 2018 and 2019

*Anopheles* species	Irrigated clusters		Non-irrigated clusters	
	Indoor	Outdoor	Indoor	Outdoor
*An. coustani* complex	0.36	2.15	0.10	0.18
*An. gambiae* s.l	3.46	3.55	0.21	0.16
*An. pharoensis*	0.24	0.27	0.06	0.05
*An. squamosus*	0.01	0.08	0.01	0.03
*An. funestus* group	0.02	0.04	0.00	0.00

**Fig. 2:**
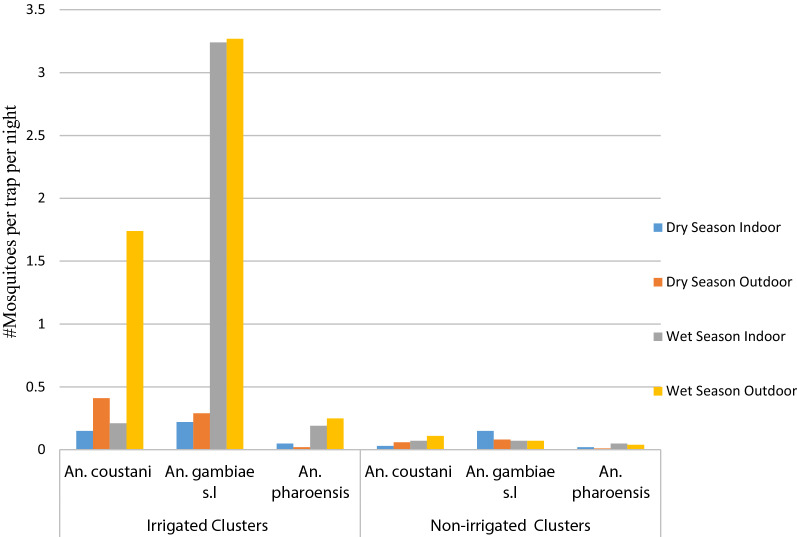
Seasonal indoor and outdoor density of malaria vectors in irrigated and non-irrigated clusters of Arjo-Didessa sugarcane irrigation scheme, Southwestern Ethiopia, 2018 and 2019

About 86% (n = 1813) of the total *Anopheles* mosquitoes were collected during the wet seasons and the remaining 14% (n = 295) collected during the dry seasons and the difference was statistically significant (χ2 = 70.423, df = 4, *P* < 0.001) (Table 3). In the wet seasons, indoor and outdoor density of *An. gambiae* s.l. was highest followed by *An. coustani* complex and *An. pharoensis* in the irrigated clusters (Fig. 2).

**Table 3:**
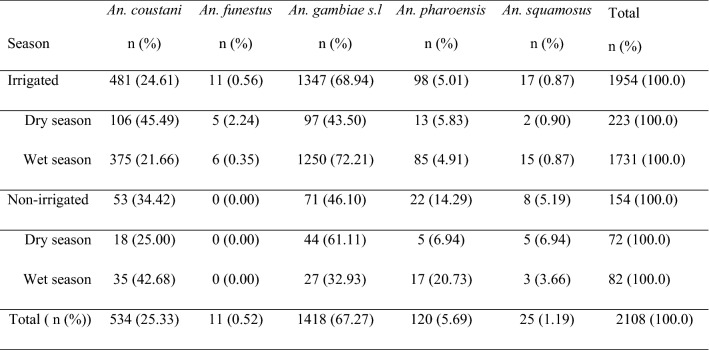
Seasonal abundance of *Anopheles* species at Arjo-Didessa Irrigation Scheme, Southwest Ethiopia, 2018 and 2019

### Mosquito species diversity

Shannon’s diversity index was 0.93 in the irrigated area and 1.15 in the non-irrigated area (Table 4). Species richness was higher in the irrigated clusters than the non-irrigated clusters. Species evenness was higher in the non-irrigated clusters than the irrigated clusters.

**Table 4:** Shannon’s diversity index of *Anopheles* species at Arjo-Didessa Irrigation Scheme, Southwest Ethiopia, 2018 and 2019

	Irrigated area	Non-irrigated area
Number of species *S*	6	4
Number of specimens *N*	1954	154
Shannon’s diversity index *H* ^’^	0.933	1.155
Evenness (*E* = *e* ^*∧*^ *H* ^’^/*S*)	0.521	0.834

## Discussion

Sugarcane irrigation activities, in Arjo-Dedissa area of southwestern Ethiopia, were associated with increased *Anopheles* species diversity, abundance and density during dry and wet seasons. Interestingly, *An. amharicus* was recorded in sugarcane irrigated areas in the present study. Indeed, irrigation provided suitable breeding grounds for malaria vector mosquitoes in the area and increased mosquito species composition and abundance. This could emanate from the availability of several and suitable *Anopheles* species breeding microhabitats as a result of the uninterrupted sugarcane irrigation activity. Six *Anopheles* species were found in the irrigated sugarcane plantation areas while only four species were collected in the non-irrigated areas. This could explain the relevance of sugarcane irrigation schemes in supporting breeding of diverse *Anopheles* species. Similar studies from Ethiopia [13] and elsewhere in Africa [20–22] suggest that irrigation agricultural practices influence *Anopheles* species diversity. The presence of such diversified malaria-transmitting *Anopheles* species might influence the risk of malaria transmission and affect vector control efforts in the irrigation scheme. However, data on sporozoite rate would be required to confirm the increased malaria risk in areas with increased vector density since elevated vector abundance does not necessary translate into increased disease risk.

Occurrence and distribution of *An. amharicus* in irrigation schemes was recorded for the first time in this study. *Anopheles amharicus* was reported for the first time in Ethiopia by Hunt et al. [23] about 18 km east of the present study area [24]. Although much is unknown about the geographic distribution of this species in Ethiopia [24], its co-existence with *An. arabiensis* in the present study indicate that these two species might have similar breeding habitat and ecologic preferences. Changes in microclimate and increased water ponding resulting from diversified habitat types, such as irrigation canals, hippo trench and man-made pools, might favour breeding and distribution of *An. amharicus* in the irrigated clusters.

The two secondary malaria vectors in Ethiopia, *An. pharoensis* and *An. funestus* group [25, 26], were also recorded in the study area, predominantly from the irrigated clusters. Similarly, these two vector species were linked with irrigation practices in Central Ethiopia [9]. A study in northern Tanzania indicate that *An. funestus* group was increased following introduction of irrigation schemes [11]. The study showed that semi-permanent ponds formed due to poorly maintained water systems were the main breeding habitats of *An. funestus* around irrigation schemes. The occurrence of diverse *Anopheles* species both in the dry and wet seasons in the irrigated clusters indicated that irrigation created conducive breeding grounds for diverse *Anopheles* species throughout most of the year. *Anopheles funestus* has become a common mosquito species in areas with water resources development in Ethiopia [9, 10].

This study clearly shows that *Anopheles* species were more abundant in the irrigated clusters than in the non-irrigated clusters both in the dry and wet seasons. Higher abundance of *An. gambiae s.l.* (primarily comprising *An. arabiensis)*, the major malaria vector, in the irrigated villages shows the role of sugarcane irrigation in increasing mosquito densities that might affect the potential risk of malaria transmission. Poorly managed irrigation creates sunlit water lodging that favour *An. arabiensis* breeding [27]. Previous studies documented *An. arabiensis* predominating in irrigated fields in Ethiopia [9, 13, 28, 29], northern Tanzania [11] and Ghana [12]. A previous study in Ethiopia also showed that an increase in canal water release to be associated with an increase in larval density of *An. arabiensis* [30]. Another study noted that *An. arabiensis* gravid females to be more attracted to sugarcane pollen-associated volatile sweet attractants [31] which might be the reason for the greater abundance of this species in the sugarcane irrigated fields in the present study. Overall, as vector abundance is one of the direct predictors for malaria transmission; this study suggested a high risk of malaria transmission around the irrigated fields unless proper vector intervention strategies are implemented.

In the present study, *Anopheles* mosquito density was generally higher outdoors than indoors, which could compromise the effectiveness of indoor-based vector interventions (long-lasting, insecticidal nets (LLINs) and indoor residual spray (IRS)). In agreement to this finding, an outdoor-biting activity of anophelines was also documented in southwestern Ethiopia [32]. This could be attributed to the intensive use of insecticide-based indoor vector control strategies (IRS and LLINs) in the area that might gradually change the mosquito feeding and resting behaviour from indoor to outdoors. Kibret and Wilson [33] noted an increasing trend of outdoor-feeding *An. arabiensis* in central Ethiopia due to extensive use of indoor insecticide-based vector interventions. In addition, presence of cattle and other animals in the vicinity that serve as an alternative source of mosquito blood meal might also contribute to the outdoor feeding tendency of anopheline mosquitoes in the study area. A targeted larval source management in the irrigated fields could help reduce vector density/abundance both indoors and outdoors [34]. Irrigation schemes should therefore consider additional vector management strategies to mitigate malaria vector breeding in such settings.

This study had several caveats. Firstly, the study lacks monthly data for adult and larval mosquito abundance. Secondly, entomological indicators such as human blood index, sporozoite rate and entomological inoculation rates were not determined. This suggests the need for further studies to confirm risk of malaria transmission. The role of *An. amharicus* in malaria transmission in the study area also requires further investigation. Research is required to evaluate the effectiveness of larval source management around irrigated schemes for mosquito control.

## Conclusion

Environmental modifications due to sugarcane irrigation schemes create conditions suitable for mosquito diversity and propagation. The increased number of malaria vector mosquitoes in the irrigated areas may increase the potential risk of malaria transmission both during the dry and wet seasons. However, further information on sporozoite rate would be required to confirm the increased malaria risk. Understanding the role of *An. amharicus* on malaria transmission in the irrigated area is important to devise tailor-made vector interventions. Current malaria vector control interventions need to incorporate larval source management to reduce vector abundance in irrigated areas.

## Data Availability

The datasets used and/or analyzed during the current study are available from the corresponding author on reasonable request.
