# Correction: Impact of sugarcane irrigation on malaria vector *Anopheles* mosquito fauna, abundance and seasonality in Arjo-Didessa, Ethiopia

**DOI:** 10.1186/s12936-023-04511-8

**Published:** 2023-03-08

**Authors:** Assalif Demissew, Dawit Hawaria, Solomon Kibret, Abebe Animut, Arega Tsegaye, Ming-Cheih Lee, Guiyun Yan, Delenasaw Yewhalaw

**Affiliations:** 1 Department of Medical Laboratory Sciences, College of Medicine and Health Sciences, Ambo University, Ambo, Ethiopia; 2 Aklilu Lemma Institute of Pathobiology, Addis Ababa University, Addis Ababa, Ethiopia; 3 Yirgalem Hospital Medical College, Yirgalem, Ethiopia; 4 School of Medical Laboratory Sciences, Faculty of Health Sciences, Jimma University, Jimma, Ethiopia; 5 College of Natural Science, Department of Biology, Jimma University, Jimma, Ethiopia; 6 Program in Public Health, University of California at Irvine, Irvine, CA 92697 USA; 7 Tropical and Infectious Diseases Research Center (TIDRC), Jimma University, Jimma, Ethiopia

## Correction: Malar J (2020) 19:344 https://doi.org/10.1186/s12936-020-03416-0

Following publication of the original article [1], it came to the authors' attention that incorrect data had been provided for Table 3. Namely, the 2nd (now 'Irrigated', but previously 'Command 5') to the 7th (now 'Wet season', but previously 'Sefera Tabiya') rows all contained incorrect data. The table has now been corrected in the published article and the corrected table can be seen in this erratum. The authors thank you for reading this correction and apologize for any inconvenience caused.

**Table 3:** Seasonal abundance of *Anopheles* species at Arjo-Didessa Irrigation Scheme, Southwest Ethiopia, 2018–2019

Season	*An. coustani* n (%)	*An. funestus* n (%)	*An. gambiae s.l* n (%)	*An. pharoensis* n (%)	*An. squamosus* n (%)	Total n (%)
Irrigated	481 (24.61)	11 (0.56)	1347 (68.94)	98 (5.01)	17 (0.87)	1954 (100.0)
Dry season	106 (45.49)	5 (2.24)	97 (43.50)	13 (5.83)	2 (0.90)	223 (100.0)
Wet season	375 (21.66)	6 (0.35)	1250 (72.21)	85 (4.91)	15 (0.87)	1731 (100.0)
Non-irrigated	53 (34.42)	0 (0.00)	71 (46.10)	22 (14.29)	8 (5.19)	154 (100.0)
Dry season	18 (25.00)	0 (0.00)	44 (61.11)	5 (6.94)	5 (6.94)	72 (100.0)
Wet season	35 (42.68)	0 (0.00)	27 (32.93)	17 (20.73)	3 (3.66)	82 (100.0)
Total (n (%))	534 (25.33)	11 (0.52)	1418 (67.27)	120 (5.69)	25 (1.19)	2108 (100.0)
